# Supplementary material for: C∙∙∙O and Si∙∙∙O Tetrel Bonds: Substituent Effects and Transfer of the SiF3 Group
Source: Int J Mol Sci. 2023 Jul 25;24(15):11884. doi: 10.3390/ijms241511884 (PMC10418337; doi:10.3390/ijms241511884)
Supplement: Supplementary file 1 [file ijms-24-11884-s001.zip › ijms-2480578-supplementary.pdf]

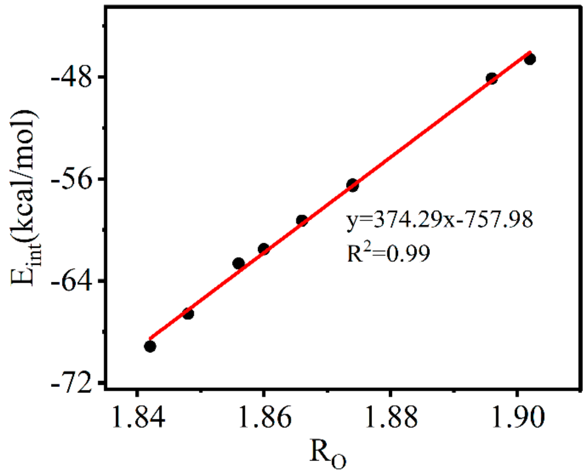

Figure S1. Linear correlation of the interaction energy (E_{int}) with the Si···O distance (R_O) in the S-SiF_3 systems

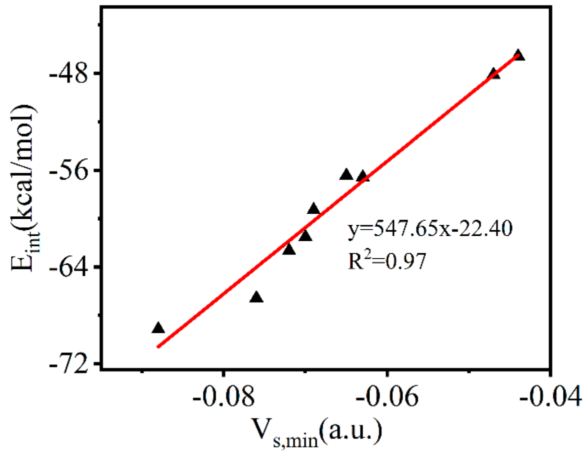

Figure S2. Linear relationship between the interaction energy (E_{int}) and the minimum MEP ($V_{s,min}$) on the O atom of POX.

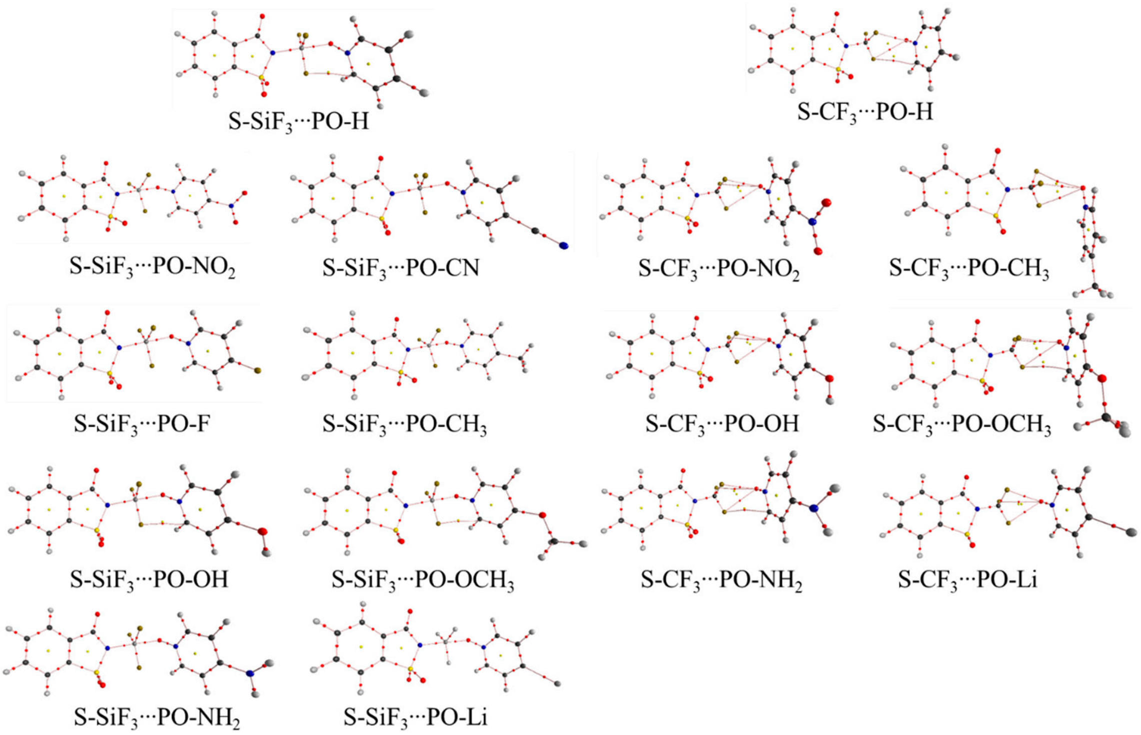

Figure S3. AIM diagrams of all complexes. Bond paths indicated by broken lines with bond critical point represented by small sphere.

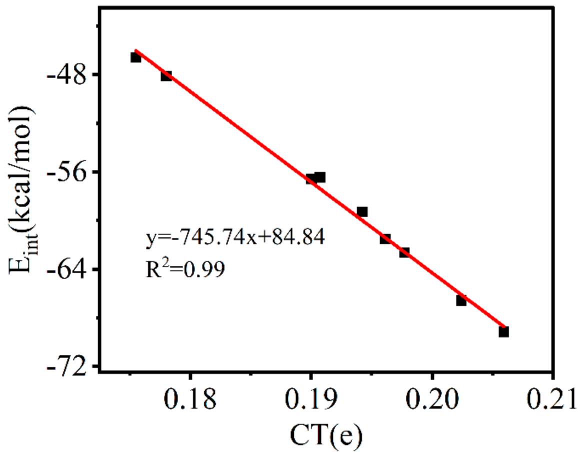

Figure S4. Linear diagram of the interaction energy (E_{int}) in relation to the charge transfer (CT) in the S-SiF_3··POX complexes

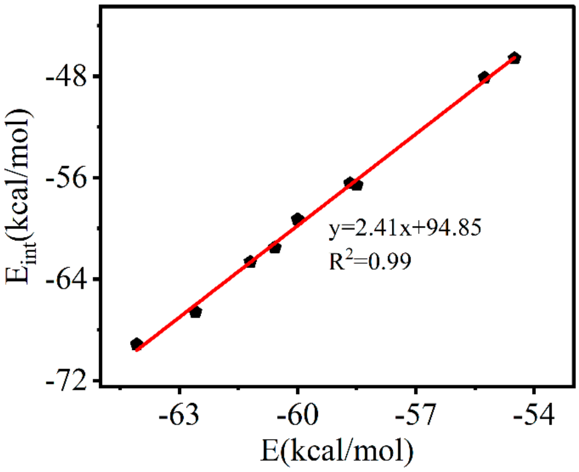

Figure S5. Linear diagram of the interaction energy (E_{int}) with the NOCV orbital energy (E) in the S-SiF_3 system

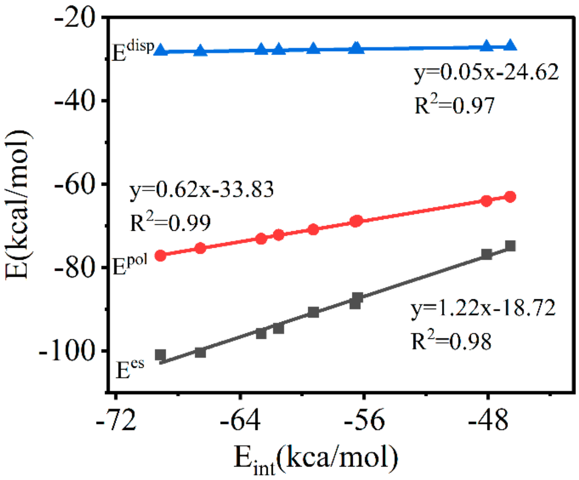

Figure S6. Linear correlation between the interaction energy (E_{int}) with three attractive terms (E^{es}, E^{pol}, and E^{disp}) from the energy decomposition of the S-SiF_3 complexes

COORDINATES OF MONOMERS AND COMPLEXES

S-CF_3

C	1.47295500	-0.50873600	-0.00539700
C	1.34053000	0.87027000	-0.02294600
C	2.47063000	1.68045200	-0.01147000
C	3.71966900	1.06117300	0.01475100
C	3.83306900	-0.33406100	0.02832100
C	2.70044100	-1.15106600	0.01762200
C	-0.07035500	1.34800000	-0.05187800
H	2.35950800	2.76318800	-0.02400800
H	4.62202600	1.66971600	0.02375800
H	4.82054800	-0.79233900	0.04857600
H	2.77699800	-2.23651500	0.02767800
N	-0.91724200	0.22997200	-0.13383800
S	-0.11498000	-1.31843300	-0.01062400
O	-0.44886100	2.48759600	-0.01659200
O	-0.32722500	-2.07406100	-1.24401900
O	-0.42177400	-1.91537800	1.28980000
C	-2.33254400	0.34953800	-0.00319800
F	-2.84081800	1.10176500	-0.97644000
F	-2.87350200	-0.86827500	-0.07354200
F	-2.67925800	0.90011100	1.16137300

S-SiF_3

C	1.75817200	-0.47728500	0.00004400
C	1.55713800	0.89384700	-0.00023800
C	2.64510600	1.76035500	-0.00027100
C	3.92318600	1.20287400	-0.00001200
C	4.10568500	-0.18550200	0.00038600
C	3.01561800	-1.05826900	0.00042500
C	0.12112300	1.28453200	-0.00013300
H	2.48160900	2.83647500	-0.00052500
H	4.79506400	1.85435500	-0.00006800
H	5.11520000	-0.59330100	0.00075600
H	3.14619300	-2.13853300	0.00077200
N	-0.68741300	0.13674900	0.00036200
S	0.20352000	-1.35370500	-0.00018300
O	-0.33041200	2.40195300	-0.00026700
O	-0.02158700	-2.04616000	-1.27086100
O	-0.02121300	-2.04743700	1.26987400
F	-2.94723000	1.04725200	-1.29558900
F	-2.97932300	-1.22039000	0.00017500
F	-2.94667000	1.04768100	1.29629700
Si	-2.42099800	0.27728500	0.00002400

PO

C	-1.09629400	-1.19240200	0.00000300
C	0.28722500	-1.17443800	-0.00002200
C	0.28721200	1.17444700	-0.00001400
C	-1.09627000	1.19242100	-0.00000200
C	-1.81659100	-0.00000600	0.00001100
H	-1.60041600	-2.15664600	0.00000400
H	0.92158700	-2.05491800	-0.00000100
H	0.92164800	2.05487400	-0.00000200
H	-1.60043000	2.15664300	-0.00000600
H	-2.90363800	0.00002400	0.00003800
N	0.98006000	-0.00002300	-0.00000700
O	2.25114200	0.00000700	0.00001900

PO-NO_2

C	-0.02757200	1.20648800	-0.00007100
C	-1.40586200	1.18298900	-0.00004900
C	-1.40578700	-1.18298500	0.00005300
C	-0.02752300	-1.20640800	0.00005700
C	0.66165500	0.00006000	-0.00002300
H	0.50930700	2.15121300	-0.00012000
H	-2.04217000	2.06165500	-0.00008500
H	-2.04210700	-2.06164200	0.00009300
H	0.50944500	-2.15108000	0.00010600
N	-2.09706300	0.00000900	-0.00000300
O	-3.35606900	-0.00005100	0.00001100
N	2.12482900	-0.00000600	0.00000000
O	2.68429900	-1.07949700	-0.00013600
O	2.68448300	1.07942000	0.00015400

PO-CN

C	0.32719300	-1.20016800	0.00002600
C	-1.05139900	-1.17918600	-0.00001900
C	-1.05152000	1.17916900	-0.00001900
C	0.32713100	1.20024900	0.00002500
C	1.04582700	0.00010000	-0.00001000
H	0.84234500	-2.15788600	0.00006600
H	-1.68480000	-2.06020400	-0.00000100
H	-1.68489700	2.06020400	0.00000100
H	0.84211300	2.15806000	0.00006700
N	-1.74489400	0.00000500	-0.00006200
O	-3.00627100	-0.00008600	0.00003900
C	2.48185800	-0.00011100	0.00001400
N	3.63930400	0.00002300	-0.00001600

PO-F

C	-0.66357000	1.20238500	-0.00000400

C	0.71984100	1.17378700	0.00000100
C	0.71983900	-1.17378900	0.00000400
C	-0.66356800	-1.20238400	-0.00000200
C	-1.35223900	0.00000200	-0.00000700
H	-1.19382900	2.15145200	-0.00000600
H	1.35194100	2.05587500	0.00000500
H	1.35194400	-2.05587200	0.00000900
H	-1.19383400	-2.15144800	-0.00000200
N	1.41202300	0.00000200	0.00000200
O	2.68679500	-0.00000200	0.00001600
F	-2.69517300	-0.00000200	-0.00001100

PO-CH_3

C	-0.60526300	1.18869600	-0.00253500
C	0.77973200	1.17044400	-0.00042000
C	0.77222800	-1.17035300	-0.00059400
C	-0.60913800	-1.18242500	-0.00231700
C	-1.34573500	0.00671700	-0.00255600
H	-1.10453400	2.15667400	-0.00453200
H	1.41087100	2.05348200	-0.00052600
H	1.40124200	-2.05495300	-0.00110200
H	-1.11401900	-2.14800400	-0.00403800
N	1.47222200	-0.00001500	0.00089700
O	2.74672600	-0.00613000	0.00235600
C	-2.85002500	-0.00204100	0.00304300
H	-3.24062400	-0.59880100	-0.83077900
H	-3.23445800	-0.43944300	0.93356000
H	-3.24863500	1.01396500	-0.08543300

PO-OH

C	0.65954100	-1.18507000	-0.00000900
C	-0.72527600	-1.17011300	-0.00002200
C	-0.74145700	1.16750600	-0.00002500
C	0.63879400	1.20636600	0.00004800
C	1.36576500	0.01609800	-0.00002300
H	1.17336500	-2.14575800	-0.00007600
H	-1.34608900	-2.06036000	-0.00000700
H	-1.37857400	2.04621700	0.00007500
H	1.15577600	2.16296400	0.00028900
N	-1.42861700	-0.00846000	-0.00008200
O	-2.70944900	-0.01687700	0.00007000
O	2.72273500	0.09625400	-0.00019800
H	3.10535100	-0.78758600	0.00150900

PO-OCH_3

C	-0.00516100	1.36377400	-0.00010400

C	1.35213200	1.13080700	-0.00001600
C	1.00151300	-1.17865300	-0.00012300
C	-0.37557100	-1.00103900	-0.00022000
C	-0.90301200	0.29018600	-0.00010500
H	-0.38063400	2.38424700	-0.00013900
H	2.10970300	1.90861900	-0.00000600
H	1.48772200	-2.14933200	-0.00007400
H	-1.00360800	-1.88668000	-0.00042000
N	1.86389200	-0.13420000	0.00004700
O	3.13101900	-0.32252000	0.00021200
O	-2.22133500	0.59814800	-0.00000200
C	-3.13500600	-0.48456200	0.00020100
H	-3.00812800	-1.10380600	-0.89942400
H	-3.00762500	-1.10406000	0.89962700
H	-4.13151900	-0.03768300	0.00061800

PO-NH_2

C	-0.63157300	-1.19178300	-0.00456500
C	0.75021700	-1.16584600	-0.00084200
C	0.74988000	1.16587100	-0.00085400
C	-0.63154300	1.19164400	-0.00462400
C	-1.36649100	-0.00023800	-0.00449300
H	-1.13405200	-2.15776400	-0.01328300
H	1.37688100	-2.05220400	0.00108400
H	1.37709900	2.05183600	0.00077500
H	-1.13446200	2.15741200	-0.01294300
N	1.44781200	-0.00018900	0.00214300
O	2.73222100	0.00029600	0.00912900
N	-2.75548700	0.00014900	-0.07159600
H	-3.20669200	-0.83929500	0.26473200
H	-3.20575400	0.84003500	0.26503600

PO-Li

C	0.92861400	-1.17003000	0.00001200
C	-0.45897300	-1.16520100	0.00001100
C	-0.45892000	1.16520800	0.00000400
C	0.92863000	1.17000400	0.00001700
C	1.71995800	-0.00004700	0.00000400
H	1.38922600	-2.16153300	0.00001900
H	-1.09016400	-2.04939700	0.00000600
H	-1.09014800	2.04937800	0.00000300
H	1.38930600	2.16147400	0.00002900
N	-1.15778800	-0.00000900	-0.00003200
O	-2.43942200	0.00004200	0.00000000
Li	3.68860300	0.00006800	-0.00004100

S-CF_3···PO

C	-2.93706400	-0.63845500	0.04845700
C	-2.92936900	0.67106800	0.49994700
C	-4.03948400	1.18917900	1.15778700
C	-5.14233700	0.35472300	1.33675300
C	-5.13305500	-0.96476000	0.86780800
C	-4.01949300	-1.48857200	0.20812400
C	-1.66296200	1.40440400	0.21572100
H	-4.02703100	2.21838800	1.51156100
H	-6.02646300	0.73272600	1.84673200
H	-6.00808800	-1.59437600	1.01911200
H	-4.00108700	-2.51164700	-0.16188600
N	-0.83997000	0.58673300	-0.57935800
S	-1.39647600	-1.06037400	-0.73944300
O	-1.37296300	2.50911500	0.58787500
O	-0.57533800	-1.92786800	0.10946400
O	-1.59247100	-1.37963200	-2.15253100
C	0.54859100	0.89393700	-0.77533000
F	1.20415800	0.93136300	0.38810400
F	1.07778800	-0.06660500	-1.53371300
F	0.68623900	2.06380300	-1.37538900
C	4.00049400	-1.90803200	0.55759300
C	3.78979500	-0.99366800	-0.45939800
C	4.20363000	0.78753200	1.01504300
C	4.42508400	-0.09887800	2.05352300
C	4.32399100	-1.47206600	1.84013700
H	3.90212100	-2.96671500	0.32812700
H	3.52948000	-1.24862600	-1.48123600
H	4.24835500	1.86867700	1.09718000
H	4.67316700	0.30436000	3.03303300
H	4.49013100	-2.17971400	2.64843600
N	3.88574900	0.34642800	-0.23426300
O	3.66102100	1.17779100	-1.17263700

S-CF_3···PO-NO_2

C	3.40202000	-0.95190700	0.22620600
C	3.61823600	0.02697500	-0.73045700
C	4.75276000	-0.01741100	-1.53263500
C	5.65265200	-1.06397500	-1.33607500
C	5.42108500	-2.04015000	-0.35921600
C	4.28106400	-2.00046500	0.44666200
C	2.55120300	1.06419300	-0.79529700
H	4.91345400	0.75333200	-2.28407200
H	6.55088200	-1.12375100	-1.94801600
H	6.14157400	-2.84482500	-0.22353700
H	4.09282700	-2.75424600	1.20857500

N	1.64169200	0.83659200	0.25370000
S	1.86969300	-0.68012600	1.09434500
O	2.45975700	1.95623200	-1.59429900
O	0.83127100	-1.62107000	0.66764700
O	2.09529600	-0.42139900	2.51506000
C	0.37172500	1.49577800	0.28248000
F	-0.34116400	1.22872300	-0.81823000
F	-0.30806100	1.04636500	1.34175300
F	0.52219800	2.80562100	0.37743400
C	-3.94214900	-0.41483700	0.98921100
C	-3.33370800	0.79972200	1.22435800
C	-3.34695500	1.30034100	-1.08742400
C	-3.95445600	0.09606800	-1.36992500
C	-4.25426800	-0.76241300	-0.31908800
H	-4.16985600	-1.08755400	1.81127100
H	-3.03774500	1.16904600	2.20047100
H	-3.06150800	2.04196600	-1.82631000
H	-4.19315400	-0.17644000	-2.39418400
N	-3.04105200	1.65661600	0.19834100
O	-2.47989600	2.76135900	0.43310000
N	-4.90375100	-2.04457700	-0.59329300
O	-5.14335600	-2.31330300	-1.75504700
O	-5.16329600	-2.75604800	0.35774300

S-CF_3···PO-CH_3

C	-3.03211800	0.87088800	0.12756500
C	-3.31521300	-0.35585900	-0.44983700
C	-4.56547300	-0.59137400	-1.01112400
C	-5.50711400	0.43618600	-0.96554900
C	-5.20368900	1.66784400	-0.37228000
C	-3.94878200	1.90804300	0.19090700
C	-2.18406400	-1.32592300	-0.40094200
H	-4.78147300	-1.55696500	-1.46456100
H	-6.49494200	0.28069400	-1.39532900
H	-5.95778200	2.45255200	-0.34864100
H	-3.70184000	2.86044300	0.65552500
N	-1.14335200	-0.76304400	0.35908200
S	-1.36510100	0.92238700	0.75264600
O	-2.14674500	-2.41233200	-0.91185200
O	-0.49443600	1.73508500	-0.10111900
O	-1.34738000	1.09667400	2.20438500
C	0.17537300	-1.33303700	0.35714700
F	0.69484900	-1.34566000	-0.87127800
F	0.94650800	-0.57521300	1.14054300
F	0.14561400	-2.56986600	0.82078100
C	4.06322000	0.56579400	-1.58030100

C	3.64856000	-0.70654200	-1.22260600
C	3.90419700	-0.17896000	1.04315600
C	4.32055300	1.09679100	0.71737200
C	4.41003900	1.51105500	-0.61570900
H	4.10727600	0.81171400	-2.63997700
H	3.35642600	-1.48296100	-1.92212400
H	3.80164200	-0.56013200	2.05410300
H	4.57283100	1.77804700	1.52888300
N	3.56387700	-1.08081800	0.08041600
O	3.15687700	-2.24867500	0.40081400
C	4.84841000	2.90421500	-0.97487100
H	4.14544900	3.64648900	-0.57602600
H	5.83820600	3.12359200	-0.55530400
H	4.89947400	3.03127000	-2.06117900

S-CF_3···PO-OH

C	3.09352000	-0.79911400	0.11409400
C	3.25950600	0.38934700	-0.57759600
C	4.44575500	0.64144000	-1.25778700
C	5.44470900	-0.33027200	-1.21272200
C	5.25940300	-1.52406400	-0.50474900
C	4.06920700	-1.78087100	0.17848100
C	2.08214300	1.30151600	-0.51034700
H	4.56955800	1.57747100	-1.79956400
H	6.38559600	-0.16041400	-1.73311200
H	6.05656300	-2.26547900	-0.48438300
H	3.91413600	-2.70430700	0.73301600
N	1.14024300	0.75335100	0.37858800
S	1.48814300	-0.88110100	0.88185400
O	1.94136400	2.33678200	-1.10261300
O	0.59613500	-1.80271500	0.17285900
O	1.59871900	-0.93670400	2.33875600
C	-0.20699400	1.24909700	0.43812200
F	-0.81915900	1.12507200	-0.74184800
F	-0.86754400	0.51957000	1.34089700
F	-0.21757300	2.52038700	0.79367100
C	-4.18850600	-1.31997900	0.56784100
C	-3.77690900	-0.08549300	1.04047200
C	-3.84287600	0.81030200	-1.11734200
C	-4.25750200	-0.39873600	-1.63923700
C	-4.43484700	-1.49271200	-0.79260800
H	-4.30767600	-2.13857500	1.27619600
H	-3.55697200	0.12786300	2.08155100
H	-3.66789700	1.70528000	-1.70620800
H	-4.43894000	-0.49735700	-2.70655700
N	-3.60090500	0.97376700	0.21162500

O	-3.19437900	2.10301900	0.66729400
O	-4.84132900	-2.66731900	-1.34283000
H	-4.88527700	-3.34922800	-0.66404500

S-CF_3···PO-OCH_3

C	-3.19152300	0.87365700	0.20735500
C	-3.38254400	-0.13778300	-0.71932600
C	-4.51259400	-0.13859800	-1.52970500
C	-5.43282700	0.89683800	-1.37045100
C	-5.22644200	1.90602900	-0.42184800
C	-4.09137400	1.91128800	0.39148600
C	-2.29319800	-1.15513200	-0.74170200
H	-4.65480200	-0.93420000	-2.25867300
H	-6.32770300	0.92145400	-1.98959300
H	-5.96296300	2.70038100	-0.31419900
H	-3.92189900	2.68923900	1.13311900
N	-1.39936000	-0.87940900	0.30941700
S	-1.66164700	0.65671500	1.09449800
O	-2.17901400	-2.06787400	-1.51386500
O	-0.63957900	1.60700600	0.64665700
O	-1.89872300	0.44896100	2.52218400
C	-0.10208700	-1.49613800	0.36147800
F	0.60191600	-1.22103600	-0.73914400
F	0.54189600	-1.00108900	1.41949100
F	-0.21416600	-2.80673000	0.47956600
C	4.03623200	0.01467100	-1.58397700
C	3.58907800	-1.20580900	-1.12985500
C	3.46384400	-0.39855900	1.05796400
C	3.90770300	0.85359500	0.65528700
C	4.19967100	1.07867500	-0.68962500
H	4.25507600	0.15546900	-2.63955700
H	3.42086300	-2.07311300	-1.76069400
H	3.20945500	-0.64921900	2.08263700
H	4.00832700	1.62627500	1.41078800
N	3.29666600	-1.41830800	0.18470200
O	2.85016600	-2.55730000	0.57468900
O	4.63301600	2.25150300	-1.20904300
C	4.76498900	3.34012000	-0.31167400
H	3.80030500	3.57553800	0.15995600
H	5.51406100	3.12063200	0.46259000
H	5.09717700	4.18833800	-0.91383200

S-CF_3···PO-NH_2

C	-3.14231100	0.73899500	0.14080700
C	-3.19566200	-0.33026000	-0.73799300
C	-4.31093400	-0.51327900	-1.54796900

C	-5.35642900	0.40309100	-1.43861900
C	-5.28607300	1.47494700	-0.54017500
C	-4.16690000	1.66240000	0.27340900
C	-1.98530400	-1.20086600	-0.70927700
H	-4.34637300	-1.35482000	-2.23731300
H	-6.24326400	0.28481600	-2.05848000
H	-6.11711200	2.17490300	-0.47343500
H	-4.10123500	2.49050600	0.97615700
N	-1.13923700	-0.75741200	0.32031900
S	-1.60803000	0.75704900	1.04593700
O	-1.75404300	-2.13280500	-1.43156200
O	-0.71607000	1.81782500	0.57042400
O	-1.83458400	0.56933900	2.47844700
C	0.21072000	-1.23667000	0.44209800
F	0.91773800	-0.96104800	-0.65573100
F	0.76983100	-0.61545500	1.48108800
F	0.21912100	-2.54287000	0.63931100
C	4.11187400	1.40692500	0.51325700
C	3.67687300	0.20715800	1.04292900
C	3.95593300	-0.85322700	-1.01504200
C	4.39992100	0.32142100	-1.59048700
C	4.49135700	1.49475700	-0.83138400
H	4.14513600	2.28065300	1.16152500
H	3.36647800	0.07280100	2.07403800
H	3.85303200	-1.79377200	-1.54703000
H	4.66790500	0.31650900	-2.64555600
N	3.59679600	-0.92113000	0.29242300
O	3.17167400	-2.02428100	0.80120200
N	4.87454500	2.70268200	-1.40300300
H	5.22142900	3.39849700	-0.75735700
H	5.44334100	2.62954100	-2.23514300

S-CF_3···PO-Li

C	-3.12093500	0.69143500	-0.04436600
C	-3.13693100	-0.61667200	-0.49833300
C	-4.30649300	-1.16081500	-1.01733500
C	-5.44148800	-0.35130100	-1.06203600
C	-5.40512000	0.96926100	-0.59826400
C	-4.23206900	1.51876900	-0.07736600
C	-1.82961900	-1.32296800	-0.36164300
H	-4.31305500	-2.19072100	-1.36967300
H	-6.37233900	-0.75030200	-1.46118300
H	-6.30616900	1.57883700	-0.64104000
H	-4.19127500	2.54326700	0.28726600
N	-0.89196900	-0.42761700	0.16722800
S	-1.51319800	1.14468400	0.57752300

O	-1.59894400	-2.46622300	-0.65317100
O	-0.88512600	2.15883000	-0.27122400
O	-1.52978100	1.30600000	2.03226000
C	0.43819600	-0.83537800	0.53700100
F	1.09331900	-1.28174400	-0.52930100
F	1.06510700	0.23342200	1.02756000
F	0.40374200	-1.79220800	1.45214400
C	4.61378700	1.58494100	-0.04531500
C	4.10452400	0.65032200	0.84477700
C	4.14775400	-0.97580400	-0.82489800
C	4.65750400	-0.04839300	-1.72226200
C	4.92227100	1.29907500	-1.39357400
H	4.76196000	2.58601400	0.36782600
H	3.85047500	0.84624900	1.88288500
H	3.92694900	-2.01486300	-1.05325700
H	4.84499900	-0.43004400	-2.72929300
N	3.86621800	-0.62920500	0.45792400
O	3.37376600	-1.48475700	1.27874000
Li	5.59425500	2.65116400	-2.65491800

S-SiF_3···PO

C	-3.16918000	-0.64135100	0.19837000
C	-3.10976900	0.73700900	0.28725300
C	-4.24697600	1.46788500	0.61239100
C	-5.43132700	0.76708500	0.83879300
C	-5.47310700	-0.62987800	0.74205200
C	-4.33064200	-1.36459400	0.41780600
C	-1.74522700	1.28347100	0.00567600
H	-4.19142500	2.55289900	0.68087200
H	-6.33947000	1.31077300	1.09394400
H	-6.41155200	-1.15179700	0.92294600
H	-4.34926400	-2.44990600	0.34020800
N	-0.86333800	0.26119300	-0.30738100
S	-1.55680600	-1.29283300	-0.21505100
O	-1.44314200	2.45554500	0.04648300
O	-1.00370600	-2.04008200	0.92589400
O	-1.57695100	-1.93111300	-1.53936600
F	1.21633800	1.21345500	0.81145900
F	1.20102700	-1.01582600	-0.81346600
F	0.60080700	1.53916300	-1.94441600
Si	0.90119900	0.58958400	-0.66947400
C	4.92023700	-1.51338100	0.32238100
C	3.99273700	-0.92463700	-0.52073300
C	3.99965500	1.04149700	0.78400700
C	4.92778400	0.49028800	1.65041300
C	5.39491900	-0.80183600	1.42129200

H	5.25408800	-2.52570000	0.11009100
H	3.54580900	-1.39148400	-1.39290300
H	3.55806600	2.02874000	0.87604200
H	5.26780800	1.07825900	2.49893300
H	6.12164600	-1.25057200	2.09577800
N	3.56568000	0.32532200	-0.26848300
O	2.69652800	0.89363100	-1.11073800

S-SiF_3···PO-NO_2

C	3.75962200	-0.95808200	-0.09844600
C	3.87897200	0.31028300	-0.63648300
C	5.05892900	0.69732100	-1.26169400
C	6.10084200	-0.22779700	-1.32065400
C	5.96188500	-1.50799900	-0.76867400
C	4.77664900	-1.89813300	-0.14231100
C	2.64471100	1.13770400	-0.46572800
H	5.14603700	1.69703400	-1.68352900
H	7.03825900	0.04552500	-1.80244500
H	6.79133100	-2.21084300	-0.82924300
H	4.65525200	-2.88923400	0.29040400
N	1.67944900	0.43442500	0.24208100
S	2.14074700	-1.16485200	0.62375400
O	2.49267500	2.26146600	-0.88837100
O	1.34580100	-2.12570900	-0.15742400
O	2.24057700	-1.34050800	2.07924600
F	-0.41216200	1.20865100	-1.01846600
F	-0.45353400	-0.25355700	1.31006200
F	0.53851100	2.42593800	1.35605100
Si	0.02448200	1.12088000	0.55585700
C	-4.26679300	0.59483700	-1.17717900
C	-3.19665100	1.33057600	-0.69959000
C	-3.19284600	0.10580300	1.32526500
C	-4.26289000	-0.66148300	0.89914200
C	-4.78397300	-0.39629400	-0.35750000
H	-4.68656600	0.78438200	-2.16087600
H	-2.68647300	2.12291100	-1.23797800
H	-2.68189000	-0.00531900	2.27637700
H	-4.67703500	-1.44454500	1.52750000
N	-2.70003600	1.07121200	0.52564700
O	-1.69577200	1.81500800	0.97451100
N	-5.93600200	-1.19454500	-0.84090500
O	-6.36926700	-2.03965900	-0.08951000
O	-6.35446000	-0.93583900	-1.94791800

S-SiF_3···PO-CN

C	-3.48693600	0.89698800	-0.07948500

C	-3.57945400	-0.38777600	-0.58232600
C	-4.76302600	-0.82841400	-1.16378000
C	-5.83623400	0.06087600	-1.21489000
C	-5.72417700	1.35834100	-0.69860700
C	-4.53510300	1.80250300	-0.11693700
C	-2.31465700	-1.17111000	-0.42461800
H	-4.82897800	-1.84104300	-1.55775000
H	-6.77782600	-0.25540500	-1.66114200
H	-6.57827700	2.03172300	-0.75061900
H	-4.43422400	2.80739900	0.28826300
N	-1.35571500	-0.42148900	0.24278300
S	-1.85568900	1.17351100	0.59109800
O	-2.13764600	-2.29925200	-0.82530600
O	-1.11308100	2.13441300	-0.24021500
O	-1.92165500	1.39024300	2.04328300
F	0.72526600	-1.14878600	-1.05905300
F	0.77697800	0.36511400	1.23788500
F	-0.11749100	-2.34535400	1.36860500
Si	0.33226600	-1.04290800	0.52565900
C	4.55652200	-0.38519100	-1.31953400
C	3.52600500	-1.15191200	-0.80775800
C	3.52331100	0.10396800	1.19220500
C	4.55269700	0.90070600	0.72651000
C	5.07865300	0.65339600	-0.54417200
H	4.94301700	-0.59577600	-2.31299100
H	3.03474800	-1.97025500	-1.32468700
H	3.03222900	0.21552800	2.15377100
H	4.93393600	1.70542700	1.34894500
N	3.04700800	-0.89303900	0.42338500
O	2.08133300	-1.66649500	0.90976200
C	6.15835500	1.46158500	-1.05080600
N	7.02935600	2.10497900	-1.45509900

S-SiF_3···PO-F

C	-3.36104000	0.77350300	-0.15311600
C	-3.38019100	-0.56848000	-0.48485000
C	-4.53590200	-1.14662500	-0.99801200
C	-5.65728800	-0.33305700	-1.15827000
C	-5.61954200	1.02443100	-0.81414800
C	-4.45876700	1.60616500	-0.30084400
C	-2.07217200	-1.25185300	-0.23518900
H	-4.54264300	-2.20361800	-1.25828300
H	-6.57873200	-0.75519600	-1.55610000
H	-6.51049600	1.63632000	-0.94838500
H	-4.41632200	2.65905900	-0.02878900
N	-1.15537800	-0.36848200	0.31415600

S	-1.74688100	1.22373000	0.46588600
O	-1.83508400	-2.41327700	-0.48246100
O	-1.06517400	2.11845500	-0.48342100
O	-1.82677400	1.61618400	1.88013400
F	0.92533200	-1.17587600	-0.91324200
F	0.93863300	0.66554400	1.14373400
F	0.18317700	-2.03683200	1.69180500
Si	0.57302900	-0.85811000	0.65566200
C	4.66584500	-0.38649500	-1.46757900
C	3.69886800	-1.08256200	-0.76819200
C	3.70593400	0.52793600	0.95684200
C	4.67312100	1.26893600	0.30483000
C	5.14235800	0.79428600	-0.91312600
H	5.03467600	-0.74990600	-2.42247500
H	3.23356400	-2.00586400	-1.09942600
H	3.24554300	0.79899400	1.90200000
H	5.04650000	2.19652200	0.72944200
N	3.25198300	-0.61575500	0.41221600
O	2.33624000	-1.33113500	1.07779100
F	6.07045400	1.48808300	-1.56479300

S-SiF_3···PO-CH_3

C	3.39307500	-0.78170300	-0.15331900
C	3.41096400	0.55790200	-0.49393700
C	4.57260400	1.13911200	-0.98979700
C	5.70135400	0.33083600	-1.12468100
C	5.66470700	-1.02477600	-0.77262400
C	4.49764300	-1.60930300	-0.27672600
C	2.09529000	1.23602400	-0.26832600
H	4.57857200	2.19446800	-1.25610400
H	6.62725700	0.75616400	-1.50827100
H	6.56077100	-1.63269900	-0.88705700
H	4.45522200	-2.66064800	0.00065700
N	1.17228600	0.34974900	0.26380300
S	1.76967600	-1.23577100	0.43798200
O	1.86088400	2.39759700	-0.51773900
O	1.11565500	-2.14436600	-0.51796000
O	1.82495900	-1.61888500	1.85674100
F	-0.91528900	1.19296400	-0.92796900
F	-0.92131100	-0.67802800	1.09958200
F	-0.12365600	2.01079800	1.67810500
Si	-0.55268600	0.85159200	0.63283100
C	-4.71462100	0.43922800	-1.38281200
C	-3.71051900	1.12251000	-0.71922300
C	-3.70838500	-0.48542200	0.99940500
C	-4.70957900	-1.19330700	0.36495700

C	-5.23623800	-0.74256000	-0.85180200
H	-5.08115800	0.83944800	-2.32512500
H	-3.23625500	2.03566300	-1.06476600
H	-3.23097400	-0.76840900	1.93212600
H	-5.07308200	-2.10898100	0.82644300
N	-3.23949100	0.64820300	0.44525900
O	-2.29313700	1.34251500	1.09116600
C	-6.31604800	-1.51632300	-1.55125600
H	-5.94793100	-2.51353800	-1.82260500
H	-7.17995300	-1.65022100	-0.88907800
H	-6.64531200	-1.00666800	-2.46128700

S-SiF$_3$···PO-OH

C	-3.36712600	-0.77501300	0.15767800
C	-3.38495400	0.56655800	0.49031500
C	-4.54121800	1.14653200	1.00009400
C	-5.66464500	0.33496800	1.15634400
C	-5.62830000	-1.02238700	0.81140900
C	-4.46689400	-1.60573000	0.30137700
C	-2.07470300	1.24778800	0.24357000
H	-4.54671100	2.20345600	1.26073100
H	-6.58663000	0.75856400	1.55145000
H	-6.52092800	-1.63265400	0.94201500
H	-4.42546600	-2.65843300	0.02839600
N	-1.15706800	0.36291800	-0.29995300
S	-1.75165200	-1.22573000	-0.45825500
O	-1.83951600	2.41020800	0.48875700
O	-1.07658400	-2.12778700	0.48927400
O	-1.83085000	-1.61516500	-1.87382000
F	0.92319000	1.18434700	0.91113000
F	0.92865900	-0.67485500	-1.13451600
F	0.16056300	2.02234700	-1.69966400
Si	0.57358200	0.85591500	-0.65649000
C	4.66824500	-1.25805000	-0.29849500
C	3.69383700	-0.53003200	-0.95052300
C	3.71275500	1.10430200	0.74345900
C	4.68679700	0.42159100	1.43694500
C	5.17576900	-0.78188800	0.91547400
H	5.01792600	-2.19074400	-0.73657900
H	3.22382000	-0.82149400	-1.88481200
H	3.25452300	2.03338600	1.06847800
H	5.06574200	0.80586200	2.37960800
N	3.24490200	0.62156500	-0.42415700
O	2.31918600	1.32952900	-1.09229100
O	6.12236800	-1.42070700	1.61977300
H	6.37325200	-2.24747100	1.19044000

S-SiF$_3$···PO-OCH$_3$

C	3.61270700	0.89626100	0.12487200
C	3.68774300	-0.40684000	0.57979600
C	4.86634200	-0.88619700	1.14037600
C	5.95317800	-0.01546700	1.22044200
C	5.85939000	1.30099700	0.75091400
C	4.67491300	1.78372000	0.19082700
C	2.41122800	-1.16631400	0.38979800
H	4.91745800	-1.91372500	1.49620700
H	6.89139200	-0.36172900	1.65136000
H	6.72431100	1.95886400	0.82176900
H	4.58821400	2.80372800	-0.17837200
N	1.45415100	-0.37143900	-0.21995000
S	1.98042600	1.21890000	-0.52597800
O	2.23202600	-2.31684700	0.72237100
O	1.27063300	2.17784000	0.33632500
O	2.04228500	1.47755100	-1.97269200
F	-0.61081800	-1.27745400	0.95832900
F	-0.66517700	0.48318700	-1.16678900
F	0.30780600	-2.18166700	-1.57807200
Si	-0.22677400	-0.99856100	-0.60993900
C	-4.44941100	-0.77235300	1.28994300
C	-3.39800300	-1.40893200	0.67662100
C	-3.42377200	0.14267700	-1.09160400
C	-4.48111200	0.82755700	-0.52184300
C	-5.00867600	0.36967700	0.69324200
H	-4.84170800	-1.14068300	2.23352600
H	-2.88501900	-2.28331000	1.06525100
H	-2.93349400	0.42683800	-2.01774600
H	-4.86379800	1.70499900	-1.03179200
N	-2.91444300	-0.94399100	-0.49462300
O	-1.91182600	-1.61298000	-1.08883300
O	-6.02261200	0.94445000	1.34429100
C	-6.60168100	2.11653800	0.77568100
H	-5.85121700	2.91303000	0.69161900
H	-7.03361900	1.89267600	-0.20854800
H	-7.38987700	2.42012500	1.46624500

S-SiF$_3$···PO-NH$_2$

C	-3.36404100	-0.78418500	0.15032700
C	-3.38595800	0.55125400	0.50555300
C	-4.54361900	1.11865500	1.02629000
C	-5.66427300	0.30083800	1.17000400
C	-5.62384100	-1.05034500	0.80190300
C	-4.46089000	-1.62094200	0.28113300

C	-2.07784400	1.24089500	0.26721800
H	-4.55228300	2.17117300	1.30425600
H	-6.58740900	0.71482100	1.57260300
H	-6.51447900	-1.66565100	0.92222300
H	-4.41603600	-2.66866400	-0.00996500
N	-1.15805800	0.36895200	-0.29126800
S	-1.74706000	-1.21770900	-0.47486400
O	-1.84883900	2.40089800	0.53051500
O	-1.07049800	-2.13496200	0.45735800
O	-1.82851400	-1.58566900	-1.89627200
F	0.91625900	1.18404500	0.92404800
F	0.92361800	-0.65127000	-1.14778900
F	0.13954500	2.05225500	-1.67420900
Si	0.57486800	0.87515000	-0.65038200
C	4.66893900	-1.23442900	-0.34270300
C	3.69909300	-0.49687400	-0.97964900
C	3.70078100	1.09571400	0.74893600
C	4.67086700	0.40079700	1.43134400
C	5.17892200	-0.80094400	0.89780100
H	5.02267900	-2.15319200	-0.80421400
H	3.23662600	-0.76988000	-1.92322100
H	3.24067500	2.01555500	1.09664800
H	5.02806900	0.78668600	2.38299000
N	3.23726900	0.64181600	-0.43042800
O	2.30443800	1.36066100	-1.08545200
N	6.14775900	-1.50117400	1.54139600
H	6.40031200	-2.42260600	1.22124200
H	6.40154600	-1.25945300	2.48614300

S-SiF_3···PO-Li

C	-3.21375900	0.72670400	-0.15533000
C	-3.21726300	-0.63757500	-0.37481700
C	-4.38325800	-1.27922400	-0.77707400
C	-5.53081700	-0.50459900	-0.94493800
C	-5.50855500	0.87751000	-0.71658800
C	-4.33721100	1.52219500	-0.31447300
C	-1.88005600	-1.27089100	-0.13694600
H	-4.37730900	-2.35426400	-0.94799700
H	-6.46076800	-0.97736900	-1.25758500
H	-6.41965400	1.45811100	-0.85466800
H	-4.30544100	2.59478200	-0.13318300
N	-0.96018600	-0.32994900	0.29277100
S	-1.57798100	1.25455100	0.33317600
O	-1.63594700	-2.44585200	-0.30232700
O	-0.97675100	2.07712200	-0.73033600
O	-1.59863300	1.78033600	1.70686800

F	1.08519100	-1.19162900	-0.94093300
F	1.12286800	0.81484600	0.96368500
F	0.43580900	-1.85168500	1.75085800
Si	0.80946400	-0.75806500	0.61446300
C	4.89338400	1.38570000	0.08959300
C	3.93149100	0.68563300	0.79987300
C	3.91840200	-0.98825400	-0.84587600
C	4.88055900	-0.29730100	-1.56520500
C	5.41982700	0.93794100	-1.14244000
H	5.22078100	2.32476600	0.53835600
H	3.48096700	0.99719100	1.73768000
H	3.45756100	-1.92799700	-1.13619600
H	5.19825600	-0.76496700	-2.49842500
N	3.46873800	-0.47735600	0.31219000
O	2.55800300	-1.16797800	1.02318100
Li	6.71876700	2.00037400	-2.21742400
